# Income assistance use among young adults who were in British Columbia special education: A longitudinal cohort study

**DOI:** 10.1371/journal.pone.0274672

**Published:** 2022-10-07

**Authors:** Craig William Michael Scott, Matthew Joseph Russell, Suzanne Tough, Jennifer D. Zwicker

**Affiliations:** 1 The School of Public Policy, University of Calgary, Calgary, Alberta, Canada; 2 Department of Medicine, Cumming School of Medicine, University of Calgary, Calgary, Alberta, Canada; 3 PolicyWise for Children & Families, Edmonton, Alberta, Canada; 4 Pediatrics, Cumming School of Medicine, University of Calgary, Calgary, Alberta, Canada; 5 Community Health Sciences, University of Calgary, Calgary, Alberta, Canada; 6 Faculty of Kinesiology, University of Calgary, Calgary, Alberta, Canada; The University of Tokyo Graduate School of Medicine Faculty of Medicine: Tokyo Daigaku Daigakuin Igakukei Kenkyuka Igakubu, JAPAN

## Abstract

**Background:**

Persons with disability (PWD) experience disproportionately high poverty rates in Canada. This trend is apparent especially among youth compared to those who develop disabilities later in life. PWD in poverty have additional needs that increase barriers to full participation in society and translate to higher basic costs for daily living. Despite the existence of income assistance programs in Canada to mitigate income inequalities faced by PWDs, access to these programs can be limited.

**Objective:**

To describe use of income assistance for young adults with disability in British Columbia for the development of potential approaches to improve realized access to these programs.

**Methods:**

We conducted a population-based retrospective cohort study using British Columbia linked administrative data. We described differences in income assistance use among PWD by the level of special education funding received during primary school education (from most to least; Level 1, Level 2, Level 3, Unfunded, and no special education) and family composition. We also provided longitudinal patterns of income assistance use.

**Results:**

Of 218,324 young adults, 88% received no special education, 0.1% used Level One, 1.6% used Level Two, 2.9% used Level Three, and 7.1% used Unfunded special education coding. Young adults with Level One special education funding had the highest rates of hospitalizations and continuing care, with no hospitalization due to homelessness. Those with Level Three special education coding had higher rates of hospitalization and hospitalization due to homelessness than Level Two young adults. When transitioning to adulthood initially, Level One and Two funded individuals used relatively more disability income assistance than individuals from the other funding levels. Nearly all BCEA users with higher funded special education codes used this disability-specific program, while lesser funded special education codes used the Temporary Assistance more frequently, for a longer duration and were more likely to be persistent Temporary Assistance users.

**Conclusions:**

Sustainable and reliable access to income assistance programs remains an issue across the heterogeneity of needs faced by young adults with disability.

## Introduction

Special education programs in Canada are designed to offer equitable educational support services for children with disabilities within environments tailored to their unique needs [1]. These programs support a range of mental health/behavioral, sensory, neurological, social, motor, and language disabilities. An estimated 546,410 (13%) of Canadian young adults aged 15–24 have disabilities [2], from which many would have received special education as children. Adult persons with disabilities (PWD) experience disproportionately higher rates of poverty than persons without disabilities in Canada [3, 4]. Youth with disabilities are among those with the highest rates of poverty, up to three times higher than those greater than 65 years of age [2]. The impact of income inequities among PWD is significant, contributing to inequities in health, disease and mortality [5]. As such, income assistance for PWD through the transition from special education to young adulthood can be an instrument to achieving greater equity. To date, there is limited research on income assistance use patterns among PWD across young adulthood.

### Persons with disability and special education

PWD are a heterogeneous population with diverse needs across the life course [2]. Activity limitations experienced by PWD are often seen throughout their daily lives, resulting in a need for services and supports [6, 7] such as special education, income assistance and other disability services to meet basic needs. The UN Convention of Rights of Persons with Disability provides guidance for signatory countries on reducing functional limitation barriers and enabling full participation in society for PWD, including these supports and services [8].

While special education in Canada offers equitable supports and services to individuals enrolled, there are others who do not realize access to these programs. It is difficult to estimate the number of Canadian children with disabilities who do not receive special education given provincial variability in education programs and the limited data published on the topic. In a 2006 national survey administered to Canadian parents, nearly half of respondents reported difficulty in accessing special education for their children with disabilities [9]. That same study estimated 40% of children with disability received special education within a given year. In the Canadian province of British Columbia (BC), among children who did not receive special education 15.6 and 19.3% were found to have diagnosed neurodevelopmental and mental health disabilities, respectively, whereas children who received special education were found to have much higher rates in both types of diagnoses (ranging from 32.9 to 78.9% by special education funding level) [7].

### Income assistance

A complex, interdependent and under-recognized relationship exists between an individual’s income and functional needs associated with disabilities [10]. Existing income assistance and disability services often consider two distinct but compounding issues: PWD face additional costs to enable participation in activities of daily living, and PWD experience higher rates of poverty and income inequality [11, 12]. Social programs are designed to address these compounding issues in Canada, where provincial governments like the BC Government provide income assistance to low-income individuals who meet eligibility criteria, including additional supports for extra costs to daily living. The British Columbia Employment and Assistance program (BCEA) serves two purposes: providing temporary, last-resort financial assistance (see reference for description of benefits) [13], to individuals who are expected to work, via the Temporary Assistance stream; and administering income assistance to PWD, via the Disability Assistance stream. To gain access to the Disability Assistance program (see reference for description of benefits) [14], an individual must receive a PWD Designation, which requires a diagnosis and professional opinion from a medical practitioner (full eligibility criteria can be found here).

### Access to income assistance programs

Sustainable and reliable access to income assistance programs is important for PWD and their families, as this funding comprises up to two-third of total income [15]. Access to these programs, however, remains an issue across the heterogeneity of needs and activity limitations among adults with disability. Policies and eligibility criteria describing access to existing income and disability supports may introduce barriers or disparities that disadvantage individuals these programs intend to assist.

The Model of Healthcare Disparities and Disability builds on the Andersen Model of access to care by adding in the individual and environmental factors impacting disparities in access to services for persons with disability (based on UN International Classification of Function for persons with disability) [16]. Importantly this model highlights the distinction between *potential access* (i.e., available programs) and *realized access* (i.e., actual use) of income assistance [16]. Changing offered program benefits and services affect potential access, while changes to eligibility, ability to navigate services, and awareness of services affect realized access. In theory, there is a need for more realized access of provincial support services to support PWD’s well-being.

### Objectives and hypothesis

Using a retrospective cohort analysis of youth with disabilities with different special education codes, we describe BCEA income assistance use over young adulthood from age 18 to 32 years old for PWD in British Columbia, Canada. This analysis aims to characterize trends in service use across this age range so areas of improved access to these programs may be identified. We expected that despite the potential access provided through the existence of income and disability assistance programs, some PWD experience lower rates of realized access to income assistance. For example, individuals with less severe disabilities may experience challenges accessing disability income assistance as they may not meet eligibility criteria.

## Methods

This study was approved through the Conjoint Faculties Research Ethics Board from the University of Calgary (REB 18–1633).

### Study design and data

We conducted a population-based retrospective cohort analysis using British Columbia linked administrative data, following STROBE reporting guidelines. Analysis described differences in income assistance utilization among PWD focusing on differences among special education coding.

BC governmental administrative data was accessed through The Data Innovation Program [17], an analytics and integration program where data is provided by government ministries for linkage and de-identification by Population Data BC [18]. Our analysis includes linked data from four BC Government ministries: Children & Family Development, Education, Health, and Social Development and Poverty Reduction [19–26]. All analysis was done through a secure portal and all reported data underwent a vetting process to ensure validity and confidentiality of the participants before leaving the secure environment. Consultation with government ministries and validity checks for each used dataset resulted in a linked longitudinal dataset from 1995 to 2017.

### Cohort

Cohort inclusion criteria required individuals to be born between April 1^st^, 1980 to March 30^th^, 1986, and for individuals to be continuously living in BC between ages 15 and 32. To establish continued residence, individuals must have had an administrative record each year from at least one of the following: BC healthcare registration, use of BCEA income assistance, appearance in the BC education system and/or the vital statistics mortality record. Mortalities through the study period were excluded, except for the analysis related to mortality numbers. A graphic representation of this longitudinal cohort is included within S1 Fig.

Special education codes were used to provide categorization regarding different disability needs and the severity of needs. Individuals within the cohort were categorized into subgroups based on the presence and level of special education funding codes. The five categories based on these codes, in descending order of education funding were Level One, Level Two, Level Three, Unfunded special education and No Special Education (Table 1). Levels One through Three receive additional funding per student (as outlined in Table 1), while all special education levels were supported by block funding provided to the school/district. To increase the number of persons enrolled in the infrequent, more severe codes to numbers sufficient for subgroup analysis, we reviewed education records for all cohort years from the 1995 fiscal year until the age of 19 years old. Further description of these funding categories can be found in the S1 File.

**Table 1:** Special education codes in British Columbia by funding levels and support amounts. Funding levels for 2016/17 are reported and are in addition to the block funding for special education.

Special Education Program	Types of Disabilities	Funding 2016/17
**Level One**	Physically Dependent, Deaf/Blind	$38,140 per student
**Level Two**	Moderate to Severe Intellectual Disability,	$19,070 per student
	Autism Spectrum Disorder (Autism),	
	Physical Disability or Chronic Health Impairment, Visual Impairment, Deaf/Hard of Hearing	
**Level Three**	Serious Mental Health Issues &	$9,610 per student
	Intensive Behaviour Interventions	
**Unfunded**	Mild Intellectual Disability,	No additional funding
	Mild Behavioural/Mental Health,	
	Learning Disability	

### Variables

Descriptive variables include relevant covariates, reported from 18 to 32 years old. Covariates were chosen based on their availability and impact on service use in other ministry systems and previous findings [6, 7]. Sex at birth and mortality (from age 18 to 32) variables were determined from BC vital statistics. The number of children-in-care, meaning they were a child in custody, care or guardianship of a person designated by the minister in 1997 or beyond [27], was determined by the presence of a case update or administrative record between 1995 and their 19^th^ birthday. High school diploma completion rates were determined based on status of receiving an accredited diploma from a BC school.

Variables describing health care service utilization were also included. The top 5% hospitalizations included any individuals who were hospitalized six or more times during the between 18 and 32 years old. These hospitalizations were derived from the Ministry of Health administrative records for the public health system, of which BC residents are a part of. Continuing care use was defined as at least one service record of Ministry of Health continuing care service between 18 and 27 years old (this was limited by data availability). Percentage hospitalized for homelessness was defined as the presence of a healthcare visit attributed, in part, to homelessness using an International Classification of Disease (ICD) code within the medical record between 18 and 32 years old. This health service variable can also provide a rough estimate of extreme homelessness within this cohort. Russell (2019) used a similar methodology for homelessness in Alberta and indicated this would only represent a small subset of the homeless population, which should be even smaller in our study as a homelessness code was not available in other available Emergency Room data, which was the majority source of health care-related homelessness reporting [6].

BCEA records occurring within a fiscal year were counted as a use of the program, and these use records included the stream used (Temporary or Disability Assistance). Individual BCEA monthly payments within the administrative record were summated per year. BCEA payments for each fiscal year were adjusted according to the BC Consumer Price Index (2019 base) to account for inflation [28]. BCEA dependents represent the size of the household sustained by the Income Assistance payment, usually comprised of spouses and children. Persistent Income Assistance use was defined as using Disability Assistance or Temporary Assistance for seven or more of the possible 15 years.

### Analysis

SAS Enterprise Guide 7.1 was used to combine the datasets using unique linkage keys provided by Population Data BC for individuals and to derive variables for the following analyses. All analysis was conducted in the Secure Research Environment provided by the Data Innovation Program.

Descriptive statistics were used to show cohort characteristics (e.g., sex, percentage of children-in-care, high school diploma completion rates, health service use) for each special education funding level and for those without special education coding. Count variables are reported as frequencies and proportions (Table 2). Proportions were calculated by dividing the number of individuals who had the attribute by the final cohort size for each subgroup.

**Table 2:** Cohort demographics by level of special education funding. Percentages are presented, with number of individuals in brackets.

	Special Education Level					
Demographic	*Level One*	*Level Two*	*Level Three*	*Unfunded*	*No Special Education*	*Entire Cohort*
*Percentage of final cohort size*, *excluding mortalities (%)*	0.1	1.6	2.9	7.1 (15581)	88.3 (192818)	218324
	(228)	(3466)	(6231)			
*Percentage of group that is male (v*. *female) (%)*	51.3	59.8	59.4	61.3	49.4	50.7
	(117)	(2073)	(3699)	(9543)	(95203)	(110637)
*Percentage who were children-in-care (%)*	11.0	9.4	32.3	7.2	1.5	2.9
	(25)	(324)	(2015)	(1121)	(2823)	(6308)
*High school diploma completion (%)*	11.0	35.6	14.8	36.0	78.7	73.0
	(25)	(1234)	(919)	(5614)	(151724)	(159516)
*Percentage in top 5% of hospitalizations (%)*	41.7	14.2	16.5	9.6	4.7	5.6
	(95)	(493)	(1030)	(1499)	(9106)	(12223)
*Percentage hospitalized for homelessness (%)*	0.00	0.9	3.0	0.8	0.2	0.3
	(0)	(32)	(190)	(124)	(350)	(696)
*Percentage required continuing care (18–27 years old) (%)*	87.3	17.8	3.3	2.3	1.4	1.8
	(199)	(616)	(204)	(364)	(2603)	(3986)
*Mortality (18 and 32 years old) (%)*	22.7	4.0	3.6	2.1	0.9	1.2
	(67)	(143)	(234)	(327)	(1801)	(2572)

Use of income assistance is reported for each special education funding level and for those without special education coding (Table 3), including: what percent used income assistance, mean duration of income assistance use, percentage that used BCEA temporary assistance and disability assistance programs, percentage of BCEA users who had two or more dependents and BCEA user family composition. Count variables are reported as frequencies and proportions.

**Table 3:** BC Employment Assistance (BCEA) use by special education level. BCEA use is described among those who use the two streams of this program, Disability Assistance and Temporary Assistance. Percentages are reported, with number of individuals in parentheses and standard deviation in square brackets.

	Special Education Level					
BCEA use pattern	Level One	Level Two	Level Three	Unfunded	No Special Education	Entire Cohort
*Percentage of group who are BCEA users*	92.5	67.9	70.3	42.8	14.8	19.3
	(211)	(2352)	(4380)	(6671)	(28615)	(42229)
*Mean duration of BCEA use in years (max 15 years)*	12.76	11.62	7.25	6.04	4.37	5.38
	[3.34]	[4.36]	[4.63]	[4.49]	[3.74]	[2.88]
*Percentage of BCEA users who ever used Temporary Assistance*	9.5	35.2	93.6	90.4	93.8	89.6
	(20)	(828)	(4101)	(6029)	(26842)	(37820)
*Percentage with Persistent Temporary Assistance Use (≥ 7 years)*	0.0	3.7	31.6	20.0	13.6	15.9
	(0)	(86)	(1385)	(1336)	(3885)	(6692)
*Percentage of BCEA users who ever used Disability Assistance*	>90*	87.4	32.6	30.1	20.6	27.5
		(2056)	(1429)	(2008)	(5891)	(11594)
*Percentage with Persistent Disability Assistance Use (≥ 7 years)*	>90*	77.3	15.6	15.0	6.9	13.4
		(1818)	(683)	(998)	(1969)	(5659)
**BCEA user family composition**	(% of 211)	(% of 2352)	(% of 4380)	(% of 6671)	(% of 28615)	(% of 42229)
*% Single Men*	47.39	45.1	27.0	24.4	29.2	24.3
*% Single Women*	45.02	26.9	9.1	9.8	13.1	13.6
*% Couples*	<8*	4.7	5.2	3.9	4.5	4.3
*% Two Parent*	<8*	6.2	9.3	10.3	9.2	10.4
*% Single Parent*	<8*	17.1	49.3	51.6	44.2	47.4
*Percentage of BCEA users who had two or more dependents*	<10*	17.1	41.3	43.6	40.8	39.8
		(402)	(1808)	(2908)	(11666)	(16794)

* The exact values were masked to reduce the risk of disclosure, as outlined in the data privacy guidelines provided by Population DataBC.

We conducted a descriptive analysis based on a group of interest in the analysis, individuals who received were persistent Temporary Assistance users (use in greater than seven of the possible 15 years). These people were chronic users of a program intended to be used as a last resort. Table 4 compares descriptive statistics between this group of individuals and other Temporary Assistance users who do not require persistent use.

**Table 4:** Characteristics of British Columbia employment assistance program—temporary assistance users by length of program use (persistent use defined as >7 years using the temporary assistance stream, half of the study period), and whether these persistent users transitioned to Disability Assistance use.

	*Non-Persistent Users (of n = 31128)*	*Persistent Temporary Assistance Users (of n = 6692)*	*Persistent Temporary Assistance Users Who Became Disability Assistance Users (of n = 1372)*
*Males (%)*	48.0	33.1	33.6
	(14950)	(2214)	(461)
*Females (%)*	52.0	66.9	66.4
	(16178)	(4478)	(911)
*Completed Diploma (%)*	40.4%	12.7	10.0
	(12556)	(849)	(137)
*Children-in-care (%)*	9.2	23.4	30.1
	(2864)	(1570)	(414)
*Hospitalized for Homelessness (%)*	1.2	3.5	7.1
	(371)	(234)	(97)
*Top 5% of Hospitalizations (%)*	13.6	26.7	37.0
	(4240)	(1790)	(508)

**Fig 1:**
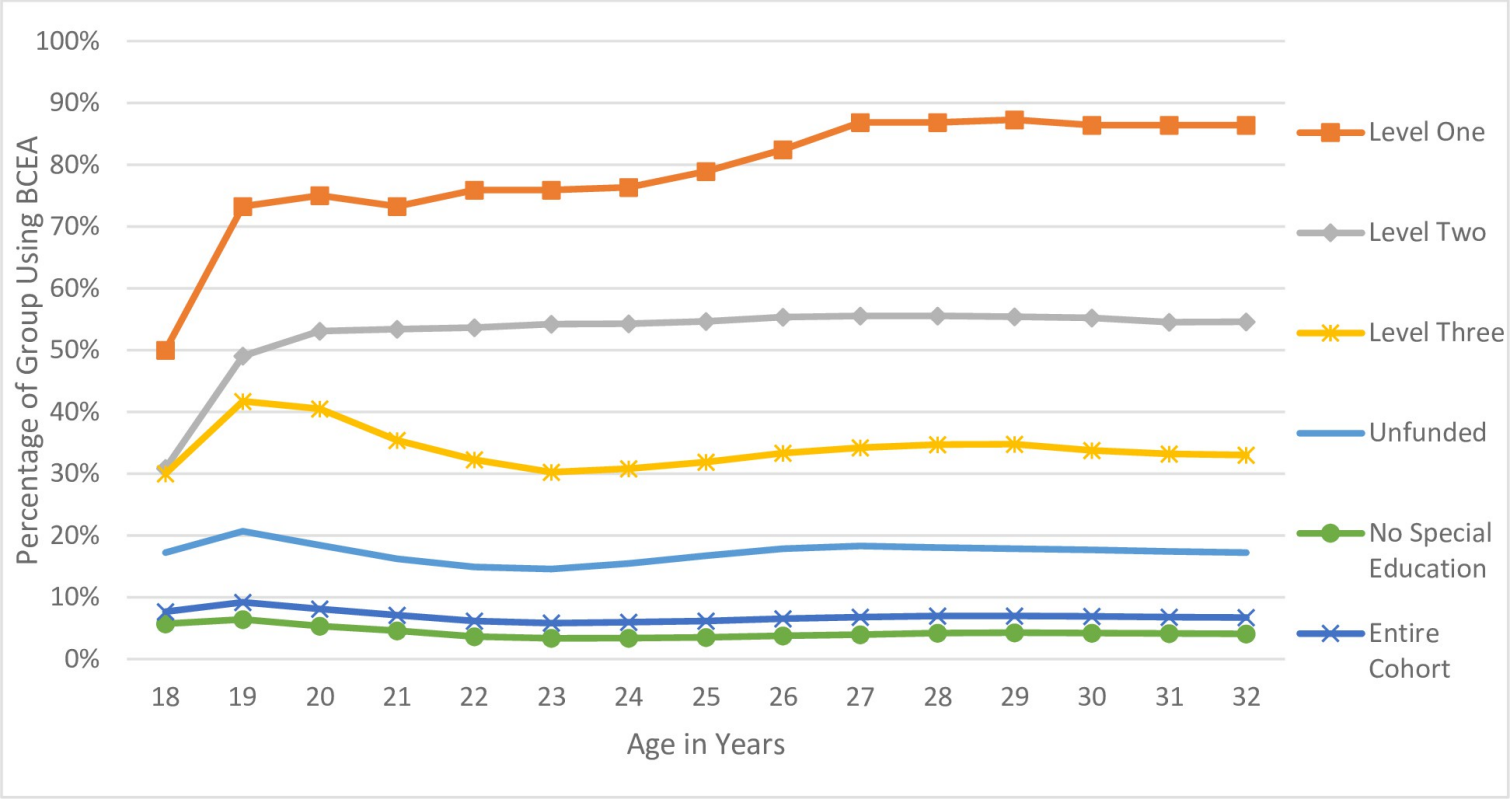
Receipt of BCEA support among those previously using special education support. Percentage of individuals within each special education group who received British Columbia Employment and Assistance program (BCEA) support during the fiscal year, by age.

**Fig 2:**
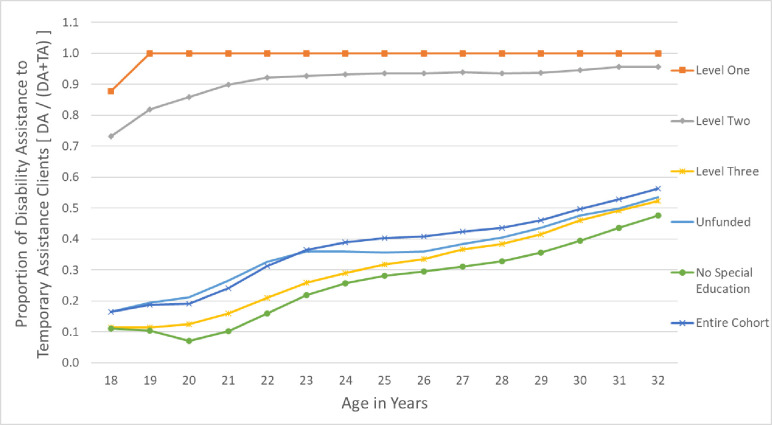
Proportion of Disability Assistance to temporary assistance clients among those previously using special education. Relative use of British Columbia Employment and Assistance–Disability Assistance (DA) program to Temporary Assistance (TA) program over young adult years, by level of special education received in primary education.

**Fig 3:**
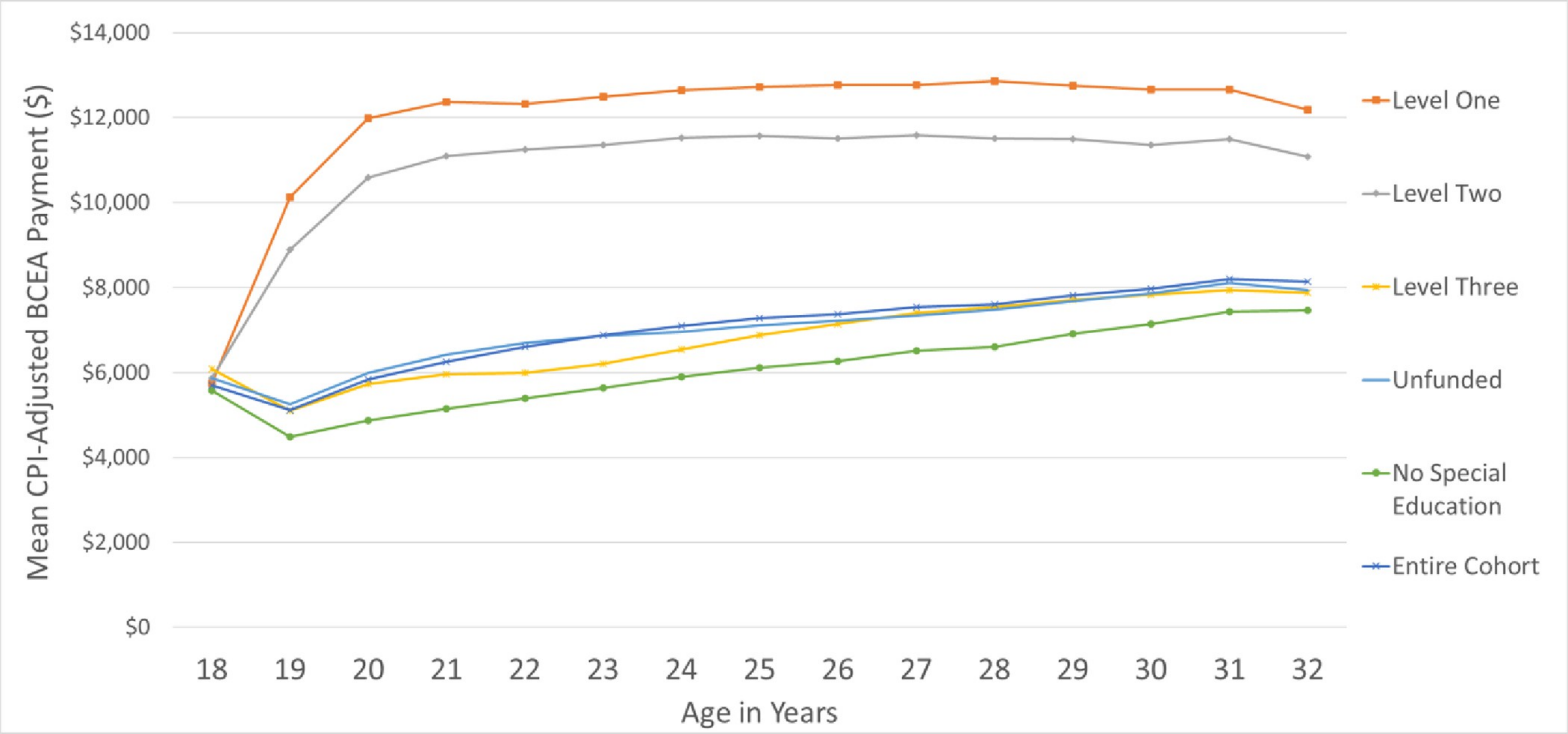
BCEA payments among those using special education. Mean inflation-adjusted British Columbia Employment and Assistance (BCEA) dollars Received over time, adjusted to British Columbia Consumer Price Index (CPI) per individual according to family equivalency scale.

To assess longitudinal use of BCEA from age 18 to 32, the mean BCEA payments received over time were calculated. The proportion of each group who were BCEA users within the year is reported in Fig 1. To control for differences in family size, we report adjusted BCEA payments using the Market Basket Measure family equivalency scale—the adjustment tool used by Canada’s Official Poverty Line to account for alternative family compositions (Fig 3). We also report the unadjusted amounts in S2 Fig. To understand differences in type of BCEA use over time, we looked at the ratio of Disability-Assistance-to-Temporary-Assistance for each special education coding type (Fig 2). Overall trends in the BCEA use for all users (including those not in the cohort) were presented for the relative use of Disability-Assistance-to-Temporary-Assistance during this period (S3 Fig).

## Results

### Demographics and outcomes

Our population-based cohort consisted of 218,324 young adults, excluding individuals that died during the period. Among these youth, 88.3% had no special education coding, and 0.1% had Level One, 1.6% had Level Two, 2.9% had Level Three, and 7.1% had Unfunded special education coding (Table 2). In each special education level, there were more males than females, despite the no special education cohort being very slightly less male than female. Mortality rates increased as the special education level of funding increased. Among those with special education coding, each level had a higher percentage of children-in-care. Among those without special education coding, 1.5% were ‘children-in-care’ in contrast to one third of youth with Level Three funding. High school diploma completion rates were also substantially lower for all special education funding levels, with 78.7% completion for those with no special education coding compared to 11.0-to-36.0% among special education students.

The care needs identified among those with Level One special education funding persisted in adulthood, with the highest rates of hospitalizations and continuing care required, with no hospitalization due to homelessness. Somewhat surprisingly, those with Level Three special education coding demonstrated higher rates of hospitalization and hospitalization due to homelessness than those in Level Two. Level Three students were 16.9 times as likely to experience hospitalization due to homelessness than those not in special education.

### BCEA income assistance use

Most individuals who had special education coding received BCEA funding at some point and used income assistance for longer than those without special education funding (Table 3). Of individuals who ever used BCEA, over 90 of Level Three, Unfunded and no special education individuals used Temporary Assistance; far higher than Level One (9.5) and Level Two (35.2) (Table 3). In S1 Table, we present BCEA use statistics for each individual special education code. The results indicate there is heterogeneity in BCEA use and type of programs within each special education funding group.

To understand income assistance needs among the cohort, we estimated the percentage of individuals who are persistently low income after age 22 for greater than 6 years. Many Level Three and Unfunded adults became persistent Temporary Assistance users, rather than Disability Assistance users. Of persistent Temporary Assistance users, nearly two thirds were female, only 12.7% completed a high school diploma, almost a quarter had been children-in-care, and more than a quarter were more hospitalized users (Table 4). Only about one fifth of these persistent users eventually received Disability Assistance.

Among the Level One and Two funding groups, the use of BCEA income assistance increased rapidly until individuals were 20 years old and then did not decrease over time (Fig 1). Level Three and Unfunded special education groups had distinct patterns in BCEA use, characterized by larger fluctuations in use. There was an initial increase in BCEA use until 19 years old, then a drop during the next few years. Temporary Assistance decreased most during this age range, especially among Level Three (Fig 2).

Many Level Three, Unfunded and no special education individuals had families and children while using BCEA supports (Table 3). A high proportion of these BCEA users were single parents. Breaking this down, we found that more than two-thirds of these single parents were female, especially during the ages of 18–22 years old. BCEA recipients who received lower-funded special education had larger families; a greater proportion had two or more dependents while using BCEA.

There were disparities in the mean amount of income assistance paid to each family from this cohort during each year of life. Level One and Two individuals received more income assistance than other individuals, that persisted into adulthood. The gap in BCEA pay between these groups decreased over time. Between 18 and 19 years old, BCEA payment amounts across the higher and lower funded subgroups crossed over (S2 Fig). We hypothesized that this pattern was due to the increased number of dependents among lower funding levels in the early years, before higher funding levels transitioned into income assistance.

BCEA dependents represent the size of the household sustained by the Income Assistance payment, usually comprised of spouses and children. Family composition accounted for the differences in funding levels in the early years, because after adjusting the BCEA payments using the Market Basket Measure family equivalency scale to account for family size, mean BCEA payments among all five groups were nearly identical at age 18 (Fig 3). However, between age 19 and 32, the mean BCEA dollars received was over $11,000 per year for those with Level One and Two special education coding, higher than the $8,000 per year for Level Three and Unfunded groups. This again in part reflects the differences in Disability Assistance vs Temporary Assistance recipients among special education funding levels, given the higher dollar value associated with Disability Assistance.

When transitioning to adulthood initially, Level One and Two funded individuals used relatively more Disability Assistance than individuals from the other funding levels. Nearly all BCEA users with higher funded special education codes used Disability Assistance (Fig 2). Over time, the use of Disability Assistance relative to Temporary Assistance changed for the other groups within our cohort. Level Three and Unfunded young adults saw the relative use of Disability-Assistance-to-Temporary-Assistance increase over time, with over half receiving Disability Assistance by age 32. This likely in part is reflecting the overall trend in the BCEA program which has gradually shifted to accepting a greater proportion of Disability Assistance users over time (S3 Fig). BCEA use between ages 20–24 roughly corresponds to changes in income assistance, like the introduction of the BCEA program in 2002.

## Discussion

Barriers to participation in education, employment, and inclusion in society result in worse outcomes for persons with disabilities, relative to other Canadians [3]. Income support programs are an important step towards reducing these barriers to participation. Having a disability inclusive design is critical to ensure the income assistance landscape in BC and other jurisdictions meet the needs of PWD. Considerations specific to PWD are critical for the overall income assistance program design, as PWD in BC comprise most of the current income assistance recipients in BC (just under 71% in 2019) [29]. Our analysis provides insight into the diverse experiences of individuals identified as having a disability in childhood through special education, and their navigation of income assistance programs in adulthood.

Our findings show the type of income assistance program use varied drastically by disability type/severity. Most individuals who received Level One and Two special education in BC become users of the Disability Assistance program before the age of 20. Level Three and Unfunded special education users accessed supports primarily by the Temporary Assistance program, which requires a more dire situation (such as virtually no other savings) and provides less supports and income to its recipients.

The BC Employment and Assistance for Persons with Disabilities Act defines a person with disabilities as an individual who is at least 18 years of age, with a severe physical or mental impairment that is expected to continue for at least two years, who is significantly restricted in his or her ability to perform daily living activities, and requires assistance with daily living activities [30]. Given the high level of needs required by these individuals with Level One and Two special education coding for daily living, their disabilities map to the eligibility criteria for Disability Assistance well. With current administrative practices, these individuals and their families must seek out and receive PWD designation to apply for Disability Assistance. To improve efficiency and avoid gaps in access to Disability Assistance (with some individuals delaying graduation or applying to Temporary Assistance first), governments could expand on “prescribed classes”, an automatic enrollment to the Disability Assistance program, for certain youth that received special education [31].

Individuals receiving Level Three and Unfunded special education are comprised of those with less visible disabilities such as mental health issues, those requiring behavioural interventions, and intellectual and learning disabilities. These disabilities are typically supported by special education later in childhood [7], as there is often a delay in presentation/recognition of disability. Parents struggle to secure special education for their children with behavioural or mental health disabilities with nearly 70% reporting challenges attaining supports, with many more reporting unmet needs [9]. The consequences of these disabilities are significant as seen by the increased hospitalization rates within the Level Three and Unfunded students. These higher rates among those with mental health disabilities were consistent with previous findings that youth with formal diagnoses have been found to have increased hospitalization rates [32].

As we have seen in our analysis, the eligibility criterion for Disability Assistance has significant implications for Disability Assistance access, particularly for some episodic or “invisible” disabilities. Findings indicate these individuals experienced challenges meeting eligibility criteria as they may have adaptive functioning needs, but do not immediately qualify for Disability Assistance. This is supported by other findings that episodic disabilities are more likely to experience greater challenges in accessing income assistance programs [33]. This has important implications for these individuals, as many are on Temporary Assistance and are experiencing low income while managing the barriers to participation in society that come with a disability. Receiving lower dollar amounts of income support than those on Disability Assistance are not the only implication of using Temporary Assistance only. Qualifying for Disability Assistance means individuals have a PWD designation. This is important because after they leave assistance (for employment or other income support programs) Disability Assistance clients maintain the health care assistance and in-kind support components of the program (not received by Temporary Assistance clients), even if their employment income rises to levels beyond the limits where they would not receive Disability Assistance financial payments.

Considering how to support individuals in special education is important as they were less likely to move on to postsecondary education, more likely to end up in corrections, and more likely to end up as reported as homeless in the health care system [6]. A key variable within the context of young adults with Level Three and Unfunded coding is whether they were a child-in-care during their developmental years. Children who were placed into foster care in BC have been shown to be ‘at-risk’ populations that are more likely to delay/not graduate, use income assistance, and to be convicted of criminal offenses [34]. Our findings indicate that a significant proportion (one third) of Level Three special education recipients were children-in-care during some point in their childhood. Young adults with the equivalent of Level Three codes in BC (severe mental health and behavioural issues in special education) or mental illness diagnosed in the health care system, experienced worse than expected health outcomes, graduation rates and mortality. An important protective factor seen in Albertan young adults in special education was getting a diploma while corrections involvement and homelessness were risk factors [6].

Many individuals with special education coding and their families have low income for extended periods and require long term use of BCEA. This is contrary to the program design goal of Temporary Assistance which does not address any disability specific considerations. At least 20% of persistent Temporary Assistance users among those with special education coding become Disability Assistance users later in life. This finding suggests there are some individuals who do not realize access to the Disability Assistance program early on and perhaps should have qualified for Disability Assistance earlier. Children-in-care, individuals who do not receive a diploma and those who receive Level Three or Unfunded special education when they were younger are all groups with high Temporary Assistance use. Challenges to realized access (actual use) to Disability Assistance can result from different priorities, mandates and approaches to service delivery across ministries (education and income assistance), inconsistent policies, distinct eligibility criteria, and a lack of data on service use provided across the continuum of care [35–37]. Future policy decisions about disability assistance programs should consider these factors.

Without access to cross ministry services, some families resort to the use of high-cost acute care services [38, 39]. For example, youth with medical complexity or behaviour challenges and disability have higher healthcare service utilization (three times more hospitalization than those without) [40, 41] and are more likely to fall in the top 5% of most frequent healthcare users [41], as seen in our study. Beyond the health system impact, we know from the literature that these families face increasing inequities such as higher financial costs, reduced work hours, and higher rates of poverty compared to others who do not have a disability [41–43].

### Limitations

A limitation of our administrative data analysis is that we were unable to capture PWD who have not accessed special education services. As such, our analysis examined access to services of those identified as eligible, rather than showing the full prevalence of PWD. Despite this limitation, the benefits of this approach was that the use of special education codes allows us to specify presence of a disability diagnosis within an administrative record, while classifying by type and severity [40, 44–46]. Another limitation of administrative data is that it suffers from data entry errors and difficulties in interpretation. For example, using services may relate to increased need or the ability to navigate the services and access them. Similarly, while we report trends like decreased Temporary Assistance users in the 20–22 age range, we do not have data related to other factors such as employment and/or graduation from postsecondary to understand factors driving BCEA use trends. Finally, this study reports trends in BCEA use among our defined cohort, but does not test the relationship between BCEA use and other service use or outcomes. This is a limitation of the descriptive statistic study design. Further research should investigate what predicts BCEA use, and the connection between services use, such as BCEA, and adult outcomes (e.g., homelessness, hospitalization, etc.) may be conducted using inferential statistics and hypothesis testing.

## Conclusions

These results provide an estimate of income assistance use by children who received special education for a range of disabilities throughout their young adult years. High rates of BCEA income assistance use were apparent for individuals who previously received any special education in BC. Students who were classified in lower funded special education categories used Temporary Assistance more frequently and for a longer duration, compared to students who were classified in higher funded special education categories who received more Disability Assistance and for a longer duration. Usage of the “last resort”, Temporary Assistance stream of income assistance in BC, by definition, means individuals who received lower-funded special education programs are impoverished for greater periods of their adult life despite the recognition of their disability in primary education. Sustainable and reliable access to appropriate designations of income assistance programs is an important issue to discuss across the heterogeneity of needs faced by young adults with disabilities. This research showcases the importance of using population-based linked administrative data to inform public policy design.

## Supporting information

S1 Fig Cohort ages, years of registration and timeframe of the cohort used in the analysis. (TIF)

S2 Fig Mean British Columbia Employment and Assistance program (BCEA) payments received over time, per family (adjusted for inflation to the 2019 British Columbia Consumer Price Index). (TIF)

S3 Fig The relative use of the two BCEA program streams- Disability Assistance (DA) and Temporary (TA)–among the users over the time. (TIF)

S1 Table BCEA use by each special education code. BCEA use is described among those who use the two streams of the program, Disability Assistance and Temporary Assistance. Percentages are reported, with number of individuals and standard deviation in brackets.
(TIF)

S1 File Categories of special education codes in the manuscript (descriptions are based on 2016/17 coding practice; derived from BC education data). (DOCX)
