# Supplementary figures and images for: Income assistance use among young adults who were in British Columbia special education: A longitudinal cohort study
Source: PLoS One. 2022 Oct 7;17(10):e0274672. doi: 10.1371/journal.pone.0274672 (PMC9543764; doi:10.1371/journal.pone.0274672)

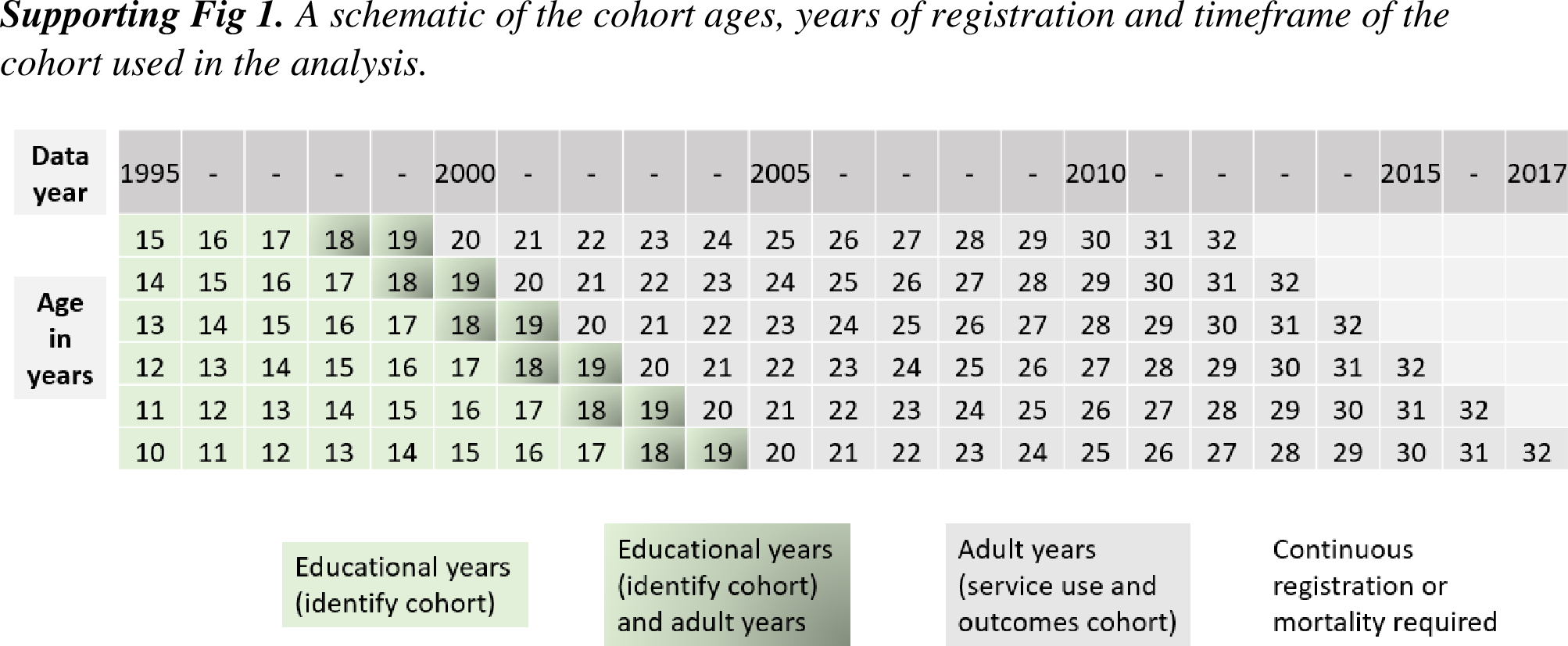

Supplement: S1 Fig — (TIF) [file pone.0274672.s001.tif]

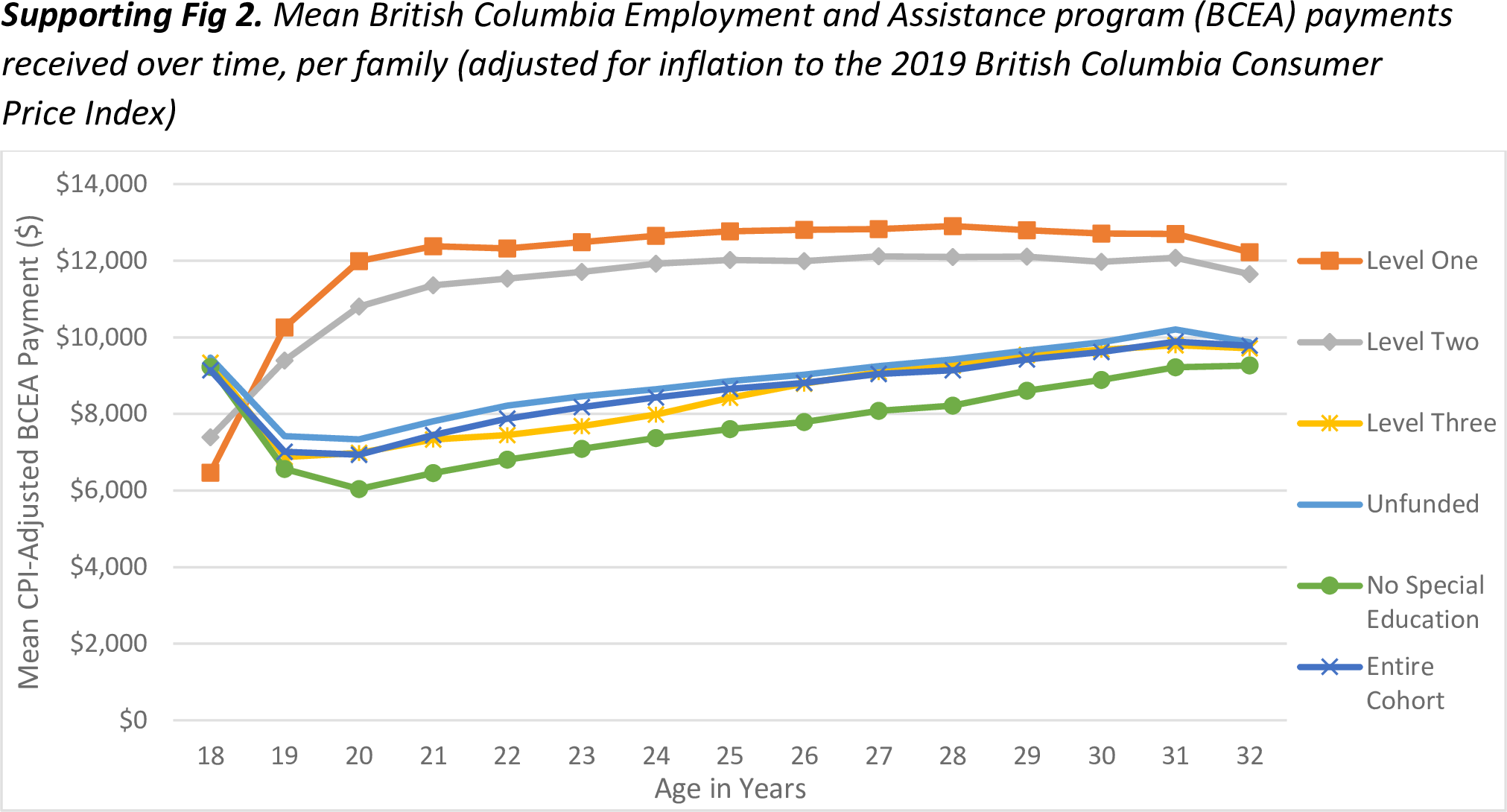

Supplement: S2 Fig — (TIF) [file pone.0274672.s002.tif]

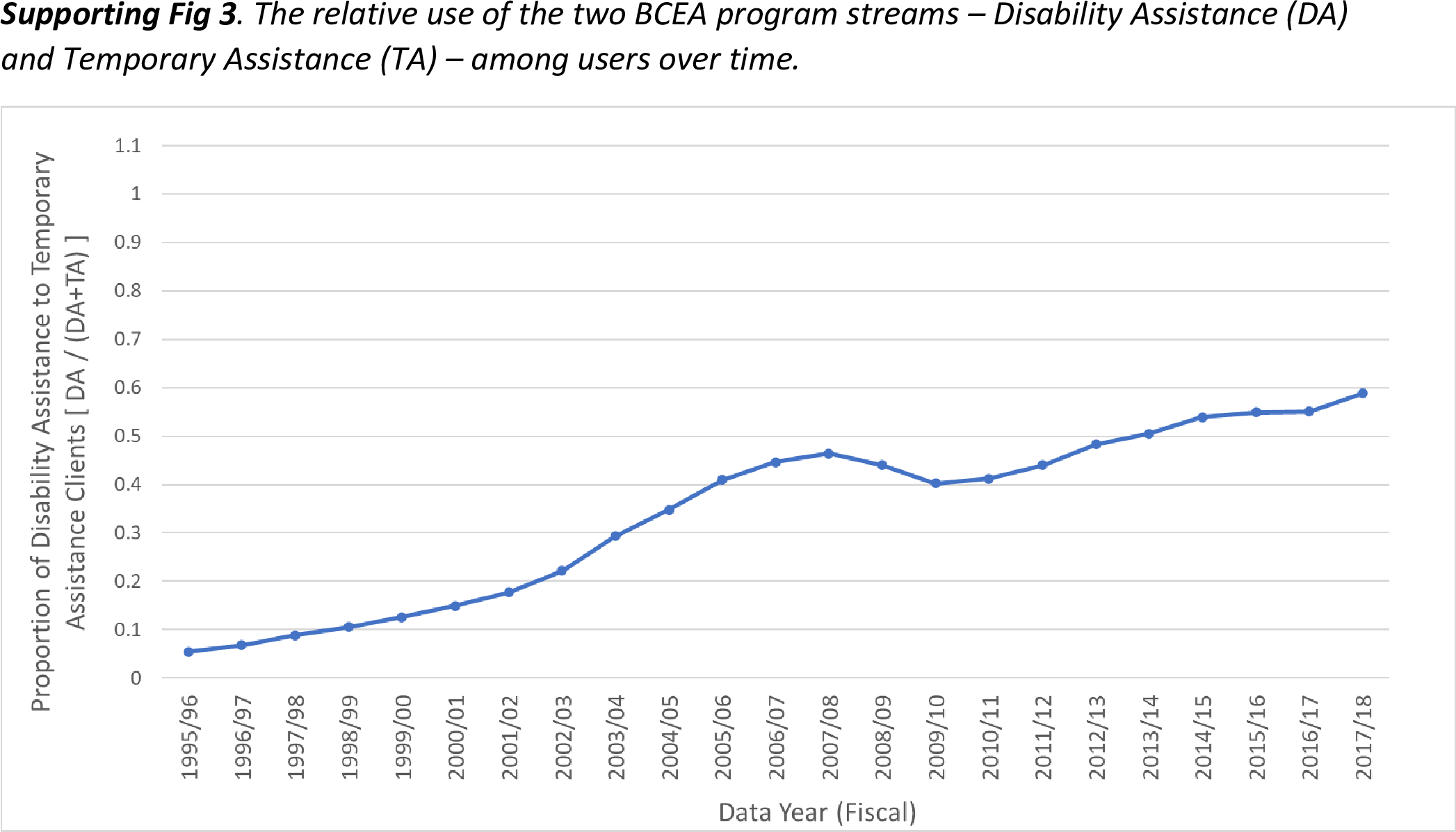

Supplement: S3 Fig — (TIF) [file pone.0274672.s003.tif]

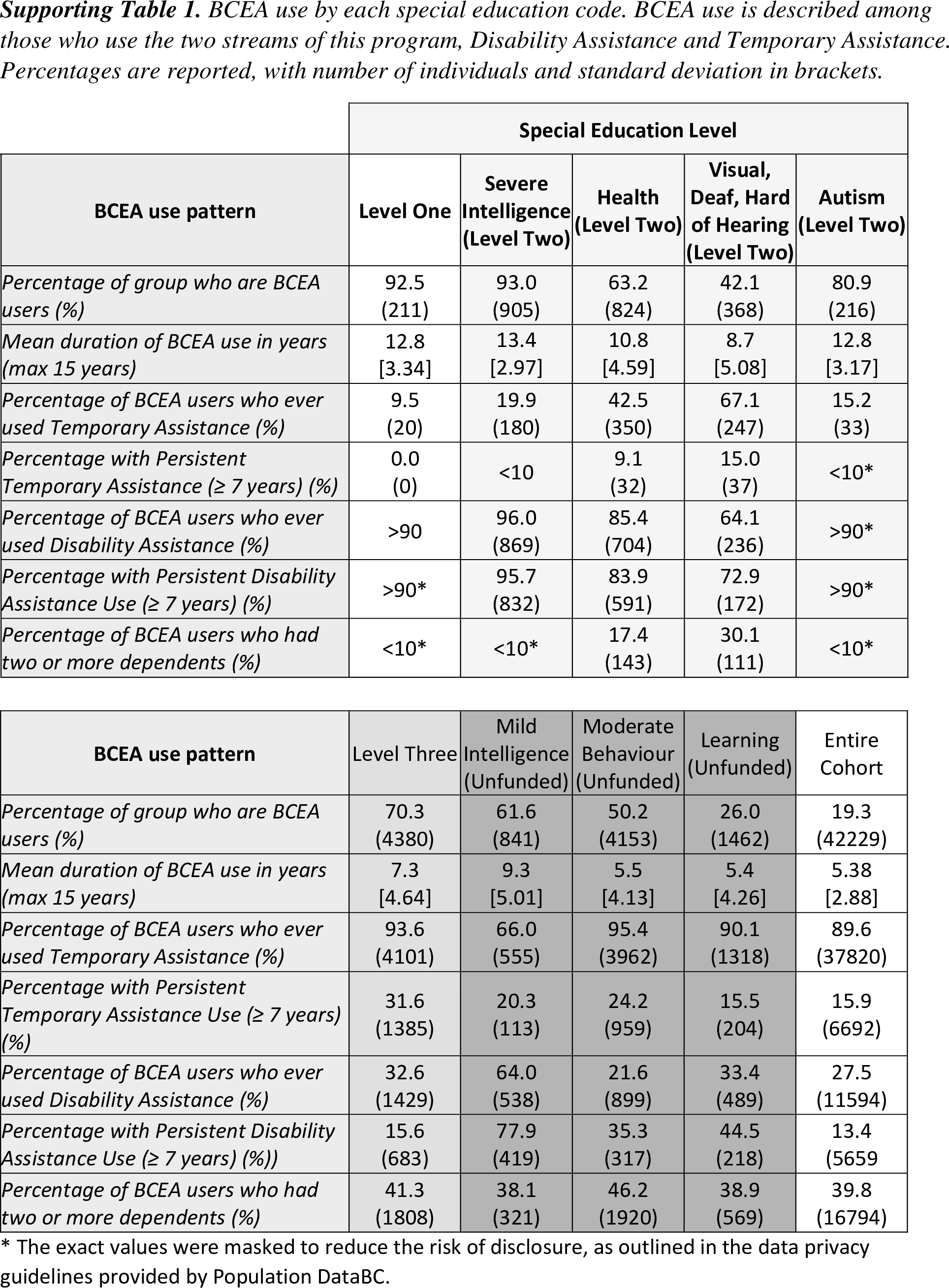

Supplement: S1 Table — BCEA use is described among those who use the two streams of the program, Disability Assistance and Temporary Assistance. Percentages are reported, with number of individuals and standard deviation in brackets. (TIF) [file pone.0274672.s004.tif]
