# Supplementary material for: Income assistance use among young adults who were in British Columbia special education: A longitudinal cohort study
Source: PLoS One. 2022 Oct 7;17(10):e0274672. doi: 10.1371/journal.pone.0274672 (PMC9543764; doi:10.1371/journal.pone.0274672)
Supplement: S1 File — (DOCX) [file pone.0274672.s005.docx]

***Supporting Material:***

**Categories of Special Education codes in the manuscript** (descriptions are based on 2016/17 coding practice; derived from BC Education data)^1^

**Level 1** (1 & 2 grouped due to low Ns)

1. **Physically dependent** (code A): The student is “completely dependent on others for meeting all major daily living needs. Requires assistance at all times for feeding, dressing, toileting, mobility and personal hygiene. Without such assistance could not attend school. May have increasing or decreasing dependence over time.”
2. **Deafblind** (code B): The student “has a degree of visual and auditory impairment which results in significant difficulties in developing communicative, educational, vocational, avocational, and social skills. Can range from partial sight to total blindness and from moderate to profound hearing loss.”

**Level 2** (3 & 4 are combined due to low Ns)

1. **Moderate to profound intellectual disability** (code C): The student has “intellectual functioning that is 3 or more standard deviations below the mean on Level C assessment instrument of intellectual functioning, and has limitations of a similar degree in adaptive functioning in at least two skill areas.”
2. **Autism spectrum disorder** (code G): Follows BC definition of Autism Spectrum Disorder, as mentioned in the Standards and Guidelines for the Assessment and Diagnosis of Young Children with Autism Spectrum Disorder in British Columbia.^2^ “Includes all of the following DSM-IV and ICD-10 categories (Autistic Disorder, PDD-NOS/Atypical Autism, Asperger Disorder/Syndrome, Rett Syndrome, and Childhood Disintegrative Disorder).” Must of had qualified diagnosis and show adverse effects on educational performance.
3. **Physical disability or chronic health impairment** (code D): The student has “nervous system impairment that impact movement or mobility, musculoskeletal condition, and/or chronic health impairments that seriously impacts student’s education and achievement.” May have different levels of need.
4. **Visual impairment** (code E): “A student with visual impairment is one whose visual acuity is not sufficient for the student to participate with ease in everyday activities. The impairment interferes with optimal learning and achievement and can result in a substantial educational disadvantage, unless adaptations are made in the methods of presenting learning opportunities, the nature of the materials used and/or the learning environment.”
5. **Deaf or hard of hearing** (code F): “A student considered to be deaf or hard of hearing is one who has a medically diagnosed hearing loss that results in a substantial educational difficulty.”

**Level 3**

1. **Intensive behavior intervention or serious mental illness** (code H): The student may require intensive behavior intervention for antisocial, extremely disruptive behaviors that are consistent/persistent over time. The student may require mental health support for serious mental illness diagnosed by qualified clinician, have serious withdrawal or internalizing behaviors, and have a history of profound problems that suggest vulnerable status. In addition, they must have serious enough challenges to be known to the school and other community agencies/service providers, pose a serious risk to self or others, and be beyond the capabilities of the school to intervene in normal capacity.

**Unfunded level**

1. **Mild intellectual** (code K): The student has “intellectual functioning that is 2 or more standard deviations below the mean on Level C assessment instrument of intellectual functioning, and has limitations of a similar degree in adaptive functioning in at least two skill areas.”
2. **Moderate behavior support or mental illness** (code M, N or R): The student may require moderate behavior support for aggression or hyperactivity, or behaviors related to social problems (e.g., delinquency, substance abuse, child abuse or neglect). The student may require mental health support for undesired psychological states (e.g., anxiety, stress-related or depression) or disabling conditions (e.g., thought disorders, neurological, or physiological). Frequency and severity must be very disruptive, be observed over an extended period of time, and not respond to normal discipline/management strategies.
3. **Learning disabilities** (code J or Q): The student has a disorder that “affects the acquisition, organization, retention, understanding or use of verbal or nonverbal information.” Must show “persistent difficulty learning, average or above average cognitive ability, and weaknesses in cognitive processing.”
